# Clinical Reports

**Published:** 1860-04

**Authors:** N. S. Davis


### CLINICAL REPORTS.

Male Ward, No. 1. Service of Prof. N. S. DAVIS. Dec., 1859.

Having frequently received letters inquiring for the results of my experience in the use of Hypophosphites and various other remedies in the treatment of Pulmonary Tuberculosis, I have thought it best to give the following brief report of a clinique on several cases of phthisis, which have been repeatedly brought to the notice of the clinical class during the past winter.

*Case* 1. Mr. B., a German, aged about 25 years, was admitted into the Hospital ten days since. At the time of his admission he had a slight fever, accompanied by soreness in his chest behind the sternum, and a pretty severe cough. He took three or four alterative doses of Hydrarg. Chlorid. Mit. with Pulv. Doveri, followed by a laxative; which lessened the heat and dryness of the skin, and somewhat relieved the soreness in the chest. But the cough and quickness of the pulse continuing, I prescribed the following mixture, to be taken in doses of a teaspoonful every four hours, viz:

℞ Comp. Honey of Squills, Senega. &c., ℥j.

Tinct. Bloodroot, ℥ss.

Camph. Tinct. Opium, ℥jss.

Tinct. Verat. Viride, ʒj. Mix.

Under the influence of this, his cough has abated but not ceased; the soreness behind the sternum has disappeared; and he has no longer any manifest febrile symptoms, except an accelerated pulse. But as you stand by his bed-side, gentlemen, you observe that his respiration is shorter and more frequent than natural; his pulse about 90 per minute and quick; his face and limbs show a moderate degree of emaciation; and he has a frequent, short cough, more severe in the morning, and accompanied with a moderate expectoration of whiteish mucus.

At the time this patient was admitted, he was undoubtedly affected with a sub-acute bronchitis. But the symptoms peculiar to that disease having subsided, while there still remains a short cough, quick sharp pulse, with moderate emaciation; the question is at once suggested whether the patient is not affected with incipient tuberculosis. The probability of this is increased by the fact that he has had more or less cough with some shortness of breath on taking muscular exercise, for three months past. But there are no symptoms on which we can rely as certainly diagnostic of tubercular disease in its early stage, except those derived from a physical examination of the chest. And even these are not among those most easy of recognition by the inexperienced. Making the chest bare, and taking one of Comman's stethescopes, we will carefully auscultate the respiration and the voice. In the infra-clavicular region of the right side we find the inspiratory murmur enfeebled and irregular in its development, while in expiration the murmur is renewed and prolonged. There is also in the same region moderate broncophony or increased vibration of voice. Over the corresponding region of the left side, the respiratory murmur is exaggerated or puerile, but neither irregular nor prolonged. After each of you have taken the stethescope and examined for yourselves, we will ascertain the result of percussion. If the room is kept perfectly still, while we percuss over corresponding parts of the two sides of the chest, you do not readily detect any alteration from the natural resonance until we reach the infra-clavicular region of the right side, where you readily recognize a moderate diminution of the resonance. Hence, we may sum up the results of the examination as follows: an enfeebled, irregular and prolonged respiratory murmur, with increased vibration of voice, and increased dulness on percussion over the infra-clavicular region of the right side; and simply an exaggeration of the respiratory murmur over the upper part of the left side. Here, you perceive, are no rhonci, or new sounds, but simple alterations of the natural ones; requiring much care to appreciate them; and yet they are of the most serious import, as has been already explained to you in the lecture room. The irregular and prolonged murmur, the moderate broncophony, and the diminished resonance, clearly demonstrate the existence of greater density than natural in the upper lobe of the right lung; while the simple exaggerated murmur of the left side is undoubtedly produced by the more forcible distension occasioned by diminished capacity of the right lung for air. But what causes the greater density of the upper lobe of the right lung? On the proper solution of this question depends the correctness of our diagnosis. We may have increased density of the lung from several different pathological conditions; from pneumonia and its consequences; from pleuritic effusions; and from tubercular deposits. The first would be preceded and accompanied by the well known phenomena of pneumonic inflammation; which have not been present during any part of the progress of the case before us. The second is always accompanied by increased *fulness* of the side affected, while the dulness is greatest in the most dependent part of the chest, instead of the upper and anterior part, as in this case. It is well-known, however, that the deposit of tubercular matter almost always commences in the upper lobe of the lung, and is accompanied by atrophy of the pulmonary tissue, instead of increased fulness. From these and many other considerations mentioned, but which would occupy too much space in this report, it is very evident that the patient before us has incipient or primary tubercular deposits in the upper lobe of the right lung. The special pathology of tubercle, and the successive changes which it undergoes, have been so fully explained to you, that we will make no comments on that subject this morning. The rational symptoms and physical signs accompanying these changes, are strikingly exhibited in two other patients in this ward.

*Case* 2. Mr. C., aged 26 years, native of Ireland, was admitted to the Hospital three days since. He has had some cough, with increasing emaciation, for the last eighteen months. You see by the vessel here that his expectoration is considerable, consisting of mucus, with circumscribed masses of distinctly purulent matter. His pulse is 100 per minute and soft; lips pale, cheeks sunken, and whole body considerably emaciated; the pulse is more frequent in the evening, with some heat of skin, and some sweating towards morning. By uncovering the chest you see the infra-clavicular space of the left side decidedly depressed, and the intercostal spaces more or less sunken on both sides. On applying the stethescope to the left infra-clavicular region, you have no respiratory murmur proper, but a loud, sharp, sub-mucous and crackling rhoncus. The broncophony is strongly marked, and so is the dulness on percussion. Here, you have all the phenomena of tuberculosis in the second or active stage of its advancement, when the tubercular masses are softening, and a slow ulcerative process is being established in the tissue surrounding them; inducing more rapid emaciation, and the slighter grade of hectic fever. To complete the examination of physical signs belonging to phthisis in the different stages of its progress, you may turn to the next bed.

*Case* 3. Mr. D., a native of Ireland, aged 30 years, has had tubercular disease for the last 3 years. You see him extremely emaciated; his pulse 110 per minute and small; respiration very short; coarse ratling of mucus in the trachea and larger bronchial tubes; voice hollow and husky; with copious purulent expectoration and night sweats. Uncovering the chest, we find the infra-clavicular region on both sides much depressed, and all the intercostal spaces sunken. On applying the stethescope to the upper lobe of the left lung, you hear a very plain cavernous sound with each respiratory act, close under the end of the stethescope, and on causing the patient to articulate sounds, he seems to speak almost directly into the end of the instrument, producing what is called pectoriloquy. Thus, gentlemen, you have in the first case examined this morning those simple alterations in the natural sounds produced by respiration, voice, and percussion, which indicate tubercular disease in its first and comparatively dormant stage.

In the second case you have all the phenomena of the second stage, or that of active softening of the tubercular masses. While in the third case, you have the cavernous respiration and pectoriloquy indicating the third stage, in which the softening has been completed, the matter discharged by expectoration, and cavities more or less numerous formed in the structure of the lung. It is not often that you can have presented to you so complete a review of the different stages of pulmonary tuberculosis in a single clinique. As the present clinique hour is exhausted, we will reserve our comments on the treatment proper for these cases until we meet in the ward to-morrow morning.

The next morning the comments on these cases were resumed in substance as follows.

Gentlemen, as we promised yesterday, your attention will be directed during the present hour to the treatment of phthisis, as suggested by the cases at present in the ward. There are but few diseases that have been subjected to a greater variety of treatment, or concerning which the professional mind has diverged into greater or more opposite extremes. It is but a few years since pulmonary phthisis was regarded as originating from inflammation, and it was deemed of the highest importance to keep each patient closely confined in a uniformly warm atmosphere; to avoid all stimulants; to restrict the diet; and to directly combat the disease by local bleeding, counter-irritation, with internal sedatives and anodynes. Subsequent investigations having developed a more correct knowledge of the pathology of tuberculosis, and clinical observations clearly proved the inappropriateness of the former treatment, at least in a large proportion of cases; the practice of the profession took so rapid a turn in the opposite direction, that the treatment advised by many at the present day might be summed up as consisting in free exercise in the open air, free use of alcoholic drinks, (especially bourbon whiskey,) cod liver oil, and the most nutritious diet. Abundant observation has satisfied me, however, that cases of tuberculosis differ much from each other in their causation or mode of development, their progress, and the co-existing condition of other important organs; and consequently that no special routine of treatment can be marked out as applicable to all cases. In many cases the development and progress of the disease is extremely slow, almost entirely exempt from inflammatory or febrile symptoms, and equally exempt from any derangements of digestion. Such cases will generally bear rich food, stimulating drinks, and abundant exercise in the open air at all seasons of the year. In another class of cases there is a low grade of tubercular inflammation in the mucous membrane of the pharynx, larynx, and bronchia, which not only greatly increases the severity of the cough, but renders the patient so sensitive to atmospheric changes that out of door exercise can be taken only to a very limited degree and with extreme caution. Still another class by no means small, presents a similar inflammatory condition of the mucous membrane of the stomach and intestines, rendering it very difficult for the patients to retain or digest anything but the most bland and unstimulating articles of diet or drink.

A well marked case of this kind now occupies a bed in the ward for females below. It is very obvious, therefore, that we cannot prescribe a fixed routine of treatment for phthisis without doing as much harm to some patients as we do good to others.

The general rules by which I am governed in the treatment of pulmonary tuberculosis, are: 1st—To give the patient as nutritious a diet as the condition of the digestive organs will bear without inconvenience. 2d—As much exercise in the open air as the strength of the patient will permit without injurious fatigue. 3d—To give such medicine as will allay the morbid sensitiveness or excitability of the respiratory organs, and improve the functions of assimilation and nutrition. 4th—To remove with the least possible waste of strength and vital power such local developments of inflammation as frequently supervene during the progress of tuberculosis.

In carrying out the first rule, many attempt to prescribe a certain amount of nutritious food, and then stimulate the digestive organs up to the point necessary for digesting it. From such a course I have never known good results.

On the contrary, I fully agree with Dr. Thomas Watson, that it is much better to adjust the quantity and quality of food to the existing condition of the stomach, than to undertake the very difficult task of adjusting the stomach to a given quantity of food. One ounce of nutritious matter *perfectly* digested and assimilated, is better for any patient than four ounces *imperfectly* prepared to nourish the textures of the body. A large proportion of phthisical patients have no difficulty in taking a sufficient quantity of any of the ordinary articles of diet, such as bread, meat, and vegetables; but for the class of patients to which I just alluded as possessing a highly irritable condition of the stomach, or what some of the older writers called "dyspeptic phthisis," the selection of diet is of paramount importance. In the great majority of such patients I have succeeded better with milk than any other article.

By adding lime-water in the proportion of one ounce to four ounces of milk, patients will generally bear from one gill to one pint at a time without inconvenience; and it contains all the elements necessary for nourishing the body more perfectly than any other one article of diet with which we are acquainted.

For drink, I induce tuberculous patients generally to use what is called "Algæ Chocolate," as a substitute for both tea and coffee. Besides being more nutritious, it contains a small proportion of iodine from the sea-weed which is mixed with the chocolate, and is therefore more or less valuable as a medicine. In regard to the patients here in the ward, the first and second cases to which I have called your attention, are able to take a reasonable quantity of all the more nutritious articles of diet. But the third case has become so much exhausted that the functions of the alimentary canal are much impaired, and he has become subject to short attacks of diarrhœa.

This patient has been obliged to rely principally on milk porrige, that is, sweet milk boiled and moderately thickened with wheat flour.

The rule in relation to exercise perhaps sufficiently explains itself. So long as the patient has sufficient strength, he should take such exercise daily as will bring the whole voluntary muscular system into action. Walking, riding on horse-back, a moderate manual labor in the open air, are the most reliable methods of exercise. The first case you have just examined, takes active exercise by walking every day; the second has too much shortness of breath to endure much walking, but might be greatly benefitted by riding in an open carriage. The third, however, is too feeble to leave his bed. To carry out the third rule requires a careful selection of such anodynes, sedatives, and tonics as are best suited to each individual case. In the early stage of the disease, while the tubercular deposit is still in its crude state, I find the majority of patients more benefited by the following remedies than any others that I have used:

℞ Fluid Ext. of Lettuce, ℥j.
Fluid Ext. of Cimicifuga, ℥j.

Mix. Give a teaspoonful before each meal and at bed-time, with five grains of Hypophosphite of Lime added to each dose when taken.

In such cases as are accompanied by passive hemorrhage, the Fluid Extract or Wine of Ergot may be substituted for the Cimicifuga with advantage. If the pulse is quick and the pulmonary organs very sensitive to atmospheric changes, much additional advantage will be derived by giving a wine-glassful of the infusion of Lycopus Virginicus or sweet Bugle half an hour after each meal. The same plan of treatment is also well adapted to some cases in the second stage of advancement. There is now in the Hospital a patient who was admitted three months since with all the symptoms of phthisis in its second stage. There was much emaciation, copious purulent expectoration, night sweats, and all the physical signs of softened tubercular disease in the upper lobes of both lungs. This patient has been kept upon substantially the same treatment as that just described. After the first six weeks the cough and expectoration began to diminish, and the latter has now ceased altogether. The hectic symptoms have also ceased, and the patient has gradually gained sufficient flesh and strength to enable him to walk about the city freely. Whether there is any truth in the theory of Dr. Churchill, or not, respecting the deficiency of phosphorous as an element in tubercular diseases, it is certain that the hypophosphites are among our best hæmostatic tonics. Still, there are some patients, even in the early stage of phthisis, who do not seem to be benefited by them. Such is the first case to which I called your attention yesterday. Previous to his admission, I several times prescribed some one of these preparations for him. But under their use his cough and other symptoms of pulmonary irritation have uniformly increased. Hence I shall keep him pretty constantly on the use of the following mixture, viz:

℞ Glycerine, ℥ jjss.

Syrup of Iodide of Iron, ℥ ss.

Sulph. Morphine, 1 gr.

Mix, and give a teaspoonful before each meal and at bed-time.

He has also taken constantly the infusion of Lycopus Virginicus after meals.* In all the advanced stages of the disease when the suppurative process is fully established, the expectoration copious, and the hectic rapidly wasting the patient, no medicine has done more in my hands to stay the progress of the disease and support the strength of the patient than this formula. Perhaps no remedy has been more generally used in the treatment of consumption during the last ten years than Cod-Liver Oil.. From much observation, I have adopted the following rule for my own patients, viz: Whenever the patient can take at least three table-spoonfuls of the oil per day, without causing nausea or impairing the relish for food, it will pretty certainly prove beneficial. But unfortunately a large majority of tuberculous patients can take it but a short time before it disturbs the stomach, so much as to do more harm than good. In those cases where it is well borne, the improvement of the patient will be rendered much more certain by giving in conjunction with the oil, five grains each of hypophosphite of Lime and Dover's Powder three times a day. In favor of the use of alcoholic drinks in the treatment of phthisis I can say nothing. I have carefully watched their influence in connection with this disease for the last five years. They are worse than useless in counteracting the tuberculous diathesis, or preventing the deposit. In the active suppurative stage of the disease their free use will sometimes retard the emacia-

* This patient now (March 28th, 1860,) is nearly free from cough or expectoration, and about his ordinary business.

tion, lessen the cough, and give a decided appearance of improvement. But it is in appearance only; for in most of such cases, while the disease of the lungs is apparently retarded, the retention of carbon in the blood hastens a fatty degeneration of the liver and kidneys, and developes dropsical effusions and albuminous urine.

Two such cases have called on me from country districts within the last three weeks. But as the clinique hour has expired, and I have previously fully discussed this subject in the lecture-room, I will not detain you longer this morning.

---

CLINIQUE OF PROF. DAVIS, IN THE MEDICAL DEPARTMENT OF LIND UNIVERSITY. Saturday, March 24th, 1860.

*Chronic Ague with Enlargement of Spleen.*—Case 1. Female child, aged 18 months. This child was first presented in the cliniques early in January last. It then presented a very bloodless appearance, with much emaciation. Its pulse was quick and feeble; skin cool; bowels a little inclined to be loose; and its appetite impaired. Its abdomen was much dis tended, partly by a very decided enlargement of the spleen, and partly by gaseous distension of the intestines.

The enlarged spleen could be easily traced by palpation and percussion. It had had regular paroxysms of intermittent fever, with only occasional short interruptions for several weeks previously. The treatment adopted consisted in the exhibition of Sulphate of Cinchonæ 1 gr. with Ferro-cyanide of Iron 1 gr. before each meal time, and a powder of Hydrarg. Cum Creta 1 gr., and Pulv. Doveri, ½ gr., every night.

No further chills occurred, and after the first four days had passed, the mercurial powder was omitted; but the Quinine and Iron have been continued with only slight interruptions until the commencement of last week. It now appears in very good health, having acquired a good degree of flesh and strength, and the evidences of splenitic enlargement having disappeared. The patient was discharged without further treatment.

Case 2. *Asthma-Chronic Bronchitis.*—This patient, a native of Ireland, aged about 50 years, first came to the Dispensary about one week since. He had been afflicted for many months with a severe harsh cough, and severe paroxysms of dyspnœa. For two weeks previous to his present visit, the cough and difficulty of breathing had been so severe each night that he had been wholly unable to lie down and sleep.

The expectoration was scanty and viscid; the skin cool; lips leaden color; and the dry bronchial rhonci easily recognized over both sides of the chest. The entire absence of febrile symptoms; the wheezing quality of the respiration; and the severe exacerbations of dyspnœa at night, plainly designated it as a case of asthma. For temporary relief at that time, he was advised to take a powder containing Pulv. Opii, 2 grs., and Tart. Ant. et Pot. ¼ gr., each morning, noon and night. He has taken these powders during the week past, and with very much relief.

The attention of the class was called to the fact that asthma, like dropsy, is merely a symptom; which is generally dependent on some prior and perhaps remote pathological condition. Thus, one class of cases depend on organic disease of the heart; another on chronic inflammation of the bronchial mucous membrane; and another on a morbid condition of the respiratory nerves, inducing purely spasmodic action. The latter cases were distinguished by the suddenness and violence of the paroxysms, and the *entire relief* from all symptoms of respiratory disturbance in the intervals. The two former are to be diagnosticated with certainty only by the aid of auscultation and percussion. Attention was called to the fact that in the present patient, there was constantly a shortness of breath, greatly increased by exercise; considerable cough, especially in the morning, with a tenacious opaque expectoration; and on applying the stethescope there was on both sides a harsh and exaggerated inspiratory murmur, somewhat prolonged in expiration, but neither broncophony nor increased dulness on percussion. The rythm and sounds of the heart were normal.

These symptoms and physical signs, existing at a time when the patient is entirely free from any special paroxysm of the asthmatic affection, are sufficient to show that the bronchial mucous membrane is thicker and more dry than natural, thereby lessening the capacity of the bronchial tubes, inducing shortness of breath, and rendering the sound produced by the ingress and egress of air harsher than natural. This state of the mucous membrane is doubtless the product of chronic inflammation. We often find similar changes in the respiratory murmur accompanying the early stage of the tubercular deposit, but this is readily distinguished by the addition of more or less broncophony and diminished resonance, symptoms that are absent in the present case.

After the students present had each made an examination of the patient with the stethescope, the following prescriptions were made for the patient, viz :

℞ Tinct. Cimicifuga Rac. ℥jss.<br>

Tinct. Lobelia, ℥ss.<br>

Tinct. Opii et Camph. ℥j.

Mix, and give a teaspoonful before each meal and at bed-time.

℞ Pulv. Aloes, 20 grs.<br>

Sulph. Ferri, 20 grs.<br>

Pillulæ Hydrarg. 10 grs.<br>

Ext. Connabis Ind. 20 grs.

Mix and divide into twenty pills. Take one pill at 8 o'clock each evening.

The patient was requested to return to the Dispensary on the next Saturday.

Case 3d.—*Chronic Bronchitis with Psoriasis.*—Male, aged 23 years, native of Ireland, laborer. This patient complained of a harsh bronchial cough, with soreness behind the sternum and in the epigastrium. He presented all the physical and rational signs of sub-acute bronchitis, resulting from a severe cold two weeks since. He also showed a well characterized patch of psoriasis on the outer part of the left arm, and a still larger patch on the leg. From the copper color of the cutaneous disease it was supposed to have some connection with a previous syphalitic influence. After explaining fully the diagnostic symptoms and nature of the cutaneous disease, the lecturer remarked that, if he was to prescribe for the bronchial affection alone, he should direct a simple anodyne and expectorant mixture, such as a combination of the Comp. Honey of Squills, Senega and Antimony, with the Camph. Tinct. of Opium. But as the present case was complicated with an obstinate cutaneous disease, dependent, in part at least, on a specific influence, he would make the following prescriptions for the present, requesting the patient to return and report progress the following week, viz:

| | | |
|---|---|---|
| ℞ | Fluid Ext. Lactuca Sat. | ℥ jss. |
| | Fluid Ext. Cinchonae, | ℥ jss. |
| | Bi Chloride Hydrarg. | 1 gr. |

Mix, and take a tea spoonful before each meal and at bed time, in a little sweetened water.

| | | |
|---|---|---|
| ℞ | Iodide of Sulphur, | ℈ i. |
| | Simple Cerate, | ℥ jss. |

Mix, thoroughly, and apply to the patches of cutaneous disease every night.

Two other cases were presented at the clinique, of which we had not time to make a memorandum.

